# Developing Infrastructure to Realize the Value of Patient-Generated Health Data in a Large Integrated Health Care System: The Veterans Health Administration Experience

**DOI:** 10.2196/70755

**Published:** 2025-06-06

**Authors:** Terry J Newton, Nilesh Shah, Katherine Lewis, Mark S Zocchi, Felicia R Bixler, Bella Etingen, Jessica M Lipschitz, Stephanie A Robinson, Timothy P Hogan, Stephanie L Shimada

**Affiliations:** 1 Office of Connected Care, Veterans Health Administration, Washington, DC, United States; 2 eHealth Partnered Evaluation Initiative, Veterans Affairs Bedford Healthcare System, Bedford, MA, United States; 3 Center for Health Optimization and Implementation Research (CHOIR), Veterans Affairs Bedford Healthcare System, 200 Springs Road, Bldg. 70, Bedford, MA, 01730, United States, 1 781-687-4737; 4 Division of Health Informatics and Implementation Science, Department of Population and Quantitative Health Sciences, University of Massachusetts Medical School, Worcester, MA, United States; 5 Center of Innovation for Complex Chronic Healthcare (CINCCH), Edward Hines Jr. Veterans Affairs Hospital, Hines, IL, United States; 6 Research and Development Service, Dallas Veterans Affairs Medical Center, Dallas, TX, United States; 7 Peter O’Donnell Jr School of Public Health, University of Texas Southwestern Medical Center, Dallas, TX, United States; 8 Department of Psychiatry, Brigham and Women's Hospital, Boston, MA, United States; 9 Department of Psychiatry, Harvard Medical School, Boston, MA, United States; 10 The Pulmonary Center, Boston University School of Medicine, Boston, MA, United States; 11 Department of Health Law, Policy, and Management, Boston University School of Public Health, Boston, MA, United States

**Keywords:** patient-generated health data, Veterans Health Administration, mobile health apps, health data interoperability, clinical decision-making

## Abstract

Patient-generated health data (PGHD) encompass health-related information created, recorded, and gathered by patients in their daily lives, and are distinct from data collected in clinical settings. PGHD can offer insight into patients’ everyday health behaviors and conditions, supporting health management and clinical decision-making. The Veterans Health Administration (VHA) has developed a robust infrastructure to collect PGHD, including automatically collected data from digital sensors and patient-entered data. This effort is guided by comprehensive policy and strategy documents to ensure the secure storage and effective use of PGHD. This paper describes the development and implementation of an infrastructure to support PGHD within the VHA and highlights envisioned clinical and research uses of PGHD to advance health care for US veterans. The PGHD database was built to Fast Healthcare Interoperability Resources standards, facilitating secure data storage and exchange of PGHD. Clinical tools, such as the provider-facing dashboards, make PGHD accessible from the electronic health records. Research and evaluation efforts focus on evaluating PGHD’s impact on patient engagement, clinical outcomes, and health care equity. The VHA’s comprehensive PGHD infrastructure represents a significant advancement in personalized health care and patient engagement. The integration of PGHD into clinical practice can enhance shared decision-making and self-management, while research and evaluation efforts can address how to maximize the benefits of PGHD for veterans. The VHA’s approach sets a benchmark for other US health care systems in leveraging PGHD to achieve the broad aims of enhancing stakeholder health care experiences, improving population health and health equity, and reducing costs.

## Introduction

Patient-generated health data (PGHD) are health-related data that are created, recorded, or gathered by patients in their everyday lives. PGHD can be used to promote health and wellness or to help address a health concern [1]. PGHD overlap with the related concepts of digital phenotyping and personal sensing but can also include nondigital format data [2,3]. PGHD are distinct from data generated in clinical settings, as they provide insight from patients’ everyday lives, outside of the health care encounter [4]. PGHD often include (but are not limited to) biometric data, symptomatology, and activity levels, and can be collected manually or through digital devices such as wearables (eg, smartwatches), mobile health apps, and Bluetooth-enabled medical devices (eg, blood pressure cuffs). Integrating PGHD into clinical care has the potential to improve outcomes by supporting care delivery processes across the care continuum, including disease prevention and diagnosis, health management, interventions, patient-provider communication, and shared decision-making [5]. In addition, PGHD have the potential to empower and engage patients in managing their own health, including engagement in healthy behaviors and monitoring of chronic conditions [6].

The Veterans Health Administration (VHA) Digital Health Office and Office of Connected Care is responsible for the development of an evolving suite of veteran- and health care team–facing virtual care tools, many of which support the collection of PGHD. In 2001, the VHA launched the tethered online patient portal, My Health*e*Vet, and later allowed veterans to manually track their personal health data, such as vital measurements, diet, sleep, or physical activity for their own records. In 2014, the VHA launched Annie for Veterans (“Annie”), a VHA SMS text messaging system modeled after the Florence Simple Telehealth system created by the National Health Service in the United Kingdom [7]. Annie sends automated self-care reminders to veterans and allows veterans to send self-entered PGHD to their clinical teams.

**Figure 1.**
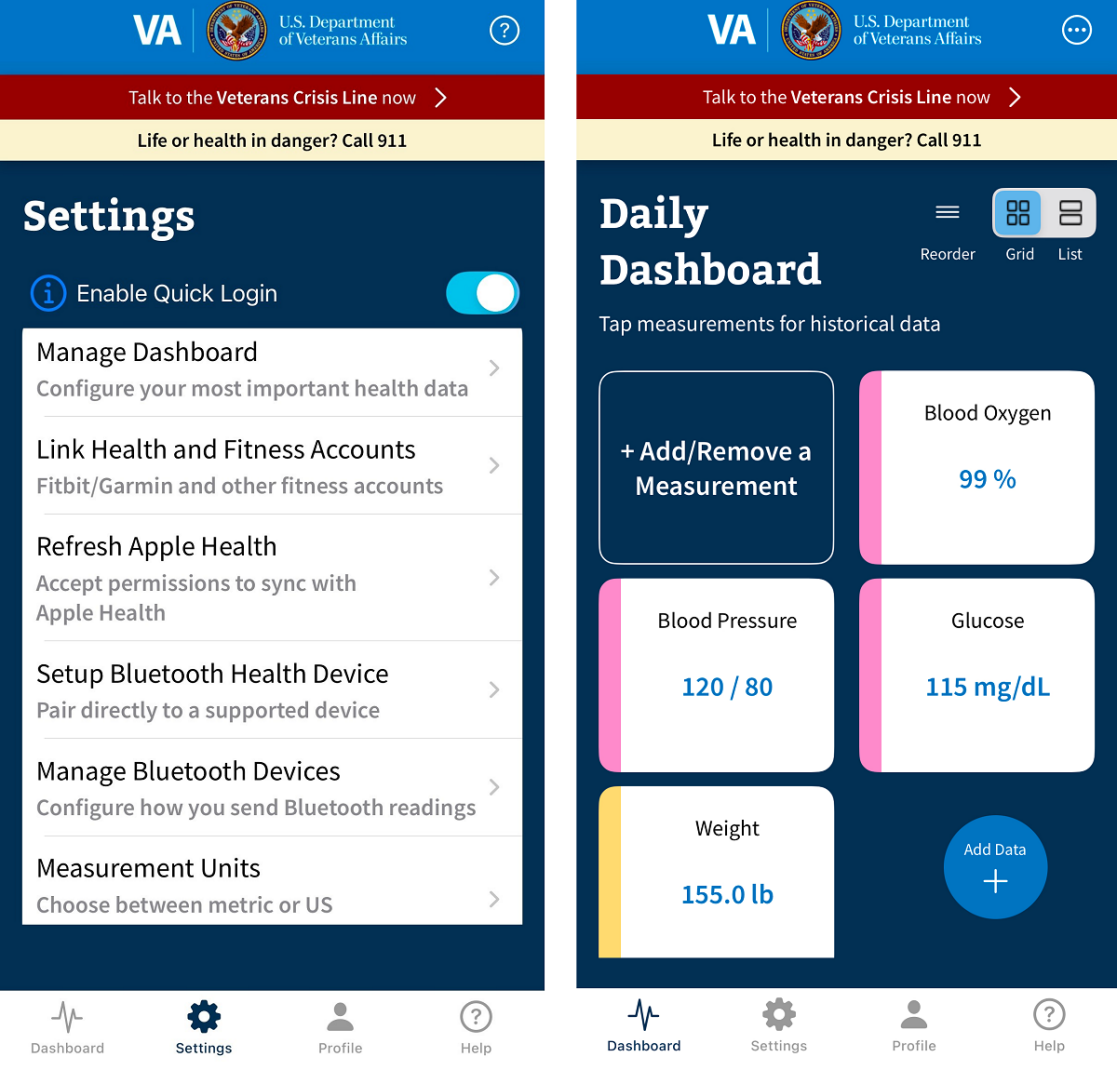
The Veterans Health Administration Share My Health Data mobile app—settings and daily dashboard screens. VA: Veterans Affairs.

In 2020, the VHA’s Office of Connected Care launched a new initiative to expand the VHA’s ability to collect PGHD from veterans who want to share their data through home medical devices (eg, digital glucometers) and activity trackers (eg, Fitbit) (Newton and Frisbee, unpublished data, 2020). In May 2023, the Office of Connected Care released the Share My Health Data mobile health app, allowing veterans to sync Bluetooth-enabled medical devices, smartwatch and wearable activity trackers, and third-party mobile health apps (eg, Apple Health) and share the corresponding PGHD with the VHA (Figure 1). The Share My Health Data app also allows veterans to directly self-enter PGHD, such as body weight, and can sync with continuous glucose monitoring devices. Prior to using the app, veterans must first accept the end-user license agreement and the data collection agreement. The data collection agreement informs the user that the VHA may collect and store any PGHD that the user has agreed to share with the VHA. Consent for the VHA to use a veteran’s PGHD includes the purposes for use such as clinical care, veteran population health initiatives, quality improvement efforts, and research.

There is great interest, both across VHA and other US health care systems, in harnessing PGHD to reduce costs, improve clinical outcomes and health equity, and enhance stakeholder health care experiences [8]. To support these goals, the VHA’s Office of Connected Care has developed the PGHD database, where veterans’ PGHD are stored and transformed into data elements that can be accessed by the VHA health care team members and researchers. The Office of Connected Care aims to support researchers who study PGHD to improve clinical care processes and veteran health outcomes, as well as integrate the collection and use of PGHD within research and evaluation projects to support their aims and objectives [1].

This manuscript describes the technical requirements, infrastructure, unique attributes and limitations, and potential clinical, research, and evaluation uses of the PGHD database as part of the VHA’s broader efforts to realize the value of PGHD in advancing health care for US veterans. As the PGHD database evolves and lessons are learned from researchers, field testers, and pilot projects, the goal will be to integrate PGHD into fundamental clinical workflows and build a knowledge base for where integration provides the greatest value.

## PGHD Technical Requirements

Data collected in the PGHD database are guided by a series of VHA policy and strategy documents, the development of which was overseen by the Office of Connected Care [9]. These documents provide guidance on key aspects of collecting, storing, and using PGHD within the health care system, including data access and management, clinical workflows, management of anticipated risks, implementation, and evaluation (U.S. Department of Veterans Affairs, unpublished data, 2021) [10]. The VHA Directive 6506 defines PGHD as, “health data created, recorded or gathered electronically by or from veterans, beneficiaries, or their authorized delegates outside the clinical health care setting to help address a health concern” [9]. The directive further defines PGHD as either solicited (ie, PGHD that the VHA providers request from veterans) or unsolicited (ie, PGHD that are provided by veterans without a request from the VHA providers) and outlines the VHA’s efforts around the use of PGHD as threefold: (1) supporting veterans with self-management; (2) supporting clinical decision-making and care delivery for individual veterans; and (3) supporting data use for health care analytics, including population health, quality improvement, and research. The expectations for provider documentation and for the use of PGHD within the electronic health record are also outlined in VHA Directive 6506.

**Figure 2.**
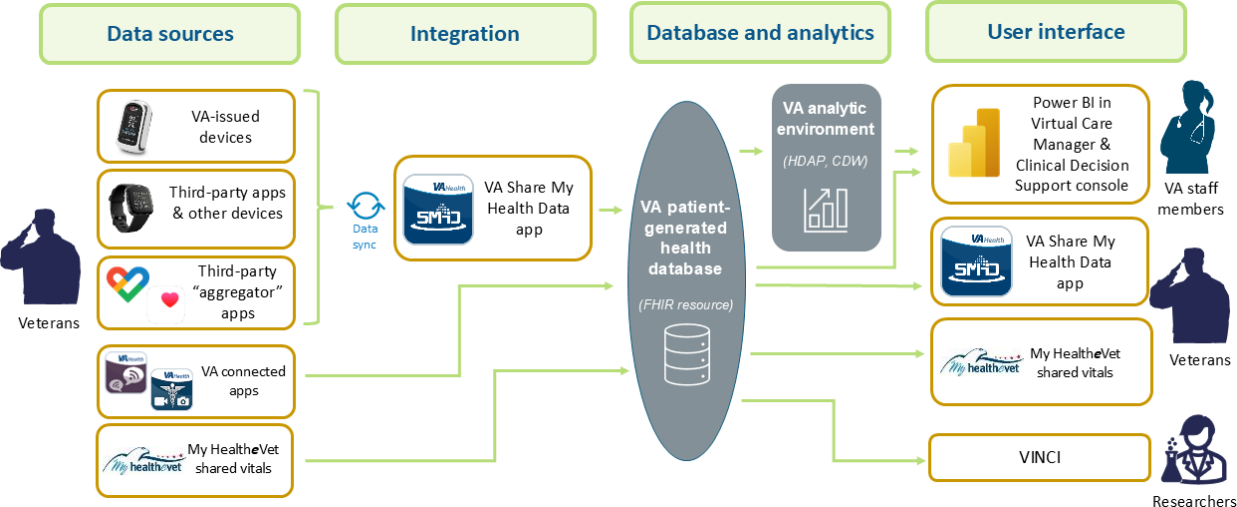
The flow of patient-generated health data (PGHD) in the Veterans Health Administration. CDW: Corporate Data Warehouse; FHIR: Fast Healthcare Interoperability Resources; HDAP: Health Data and Analytics Platform; VA: Veterans Affairs; VINCI: VA Informatics and Computing Infrastructure.

The VHA’s PGHD database contains data collected from several sources, including VHA mobile apps, third-party mobile health apps, and Bluetooth-enabled digital devices. Figure 2 depicts the data flow from these sources to the end-users of the data. To ensure the standardization across sources, data in the PGHD database go through a Fast Healthcare Interoperability Resources (FHIR)–compliant application programming interface (API). The FHIR standard is designed to facilitate the exchange of data between different health care applications by organizing data elements into resources and transmission through an API [11]. PGHD obtained through the API are stored in an analytic cloud environment via two processes: one is a direct copy of the FHIR-compliant data (in JavaScript Object Notation [JSON] format); and the other is a normative copy created by an Extract, Transform, and Load data integration process. Once loaded into the Corporate Data Warehouse and the Health Data and Analytics Platform, data can be displayed for viewing by VHA clinicians and staff through the Virtual Care Manager and Clinician Decision Support Consoles, and by veterans through the Share My Health Data App. A parallel copy is loaded on the VHA’s Informatics and Computing Infrastructure Workplace servers for operational and research use. Separate data integration and validation processes are applied to other sources, so that these data sources can also be stored in the PGHD database using the same FHIR standard.

## PGHD Infrastructure

A formal PGHD governance structure ensures that data contained in the PGHD database adhere to the FHIR data standard and mappings, and that data are stored securely and in accordance with VHA policy. Rows in the database represent observations of specific PGHD measurements taken over a specific time period, such as the number of steps taken during a calendar day, systolic blood pressure taken at a specific date and time in mm Hg, or total sleep duration in minutes. Data elements in the PGHD are linked to specific patients in the VHA through a unique patient identifier and can be merged with other VHA data sources in the VHA’s Corporate Data Warehouse. Across data sources, over 130 million observations of PGHD have been collected from over 25,000 patients in the VHA since May 1, 2023. While the PGHD database contains data from Annie and patient-entered vital signs, most of the observations come from third-party health apps connected via the Share My Health Data App. Share My Health Data allows users to share data collected from health aggregator apps such as Apple Health, fitness trackers such as Fitbit or Garmin, pair over 50 Bluetooth-enabled medical devices, and input patient-entered vitals. The most common types of PGHD are from daily activity summaries (eg, daily steps and distance traveled), exercise, sleep, and vital signs. Recent updates enabling the collection of high-frequency data from digital sensor devices (eg, minute-by-minute heart rate [pulse] data, glucose readings from continuous glucose monitoring devices, etc) will allow even more granular insight into veterans’ experiences outside the clinical encounter.

Importantly, PGHD from clinical programs, such as remote home monitoring telehealth, are currently not stored in the PGHD database. These programs are closely monitored by clinicians through established protocols and procedures. Conversely, data contained in the PGHD database are more likely to fall under the category of “unsolicited” data and will not typically be actively monitored. Providers retain autonomy in determining with their patients when to review unsolicited PGHD and are not responsible for making use of the data unless doing so is specified in the veteran’s care plan [9].

## Unique Attributes and Potential Limitations of Unsolicited PGHD

Although PGHD can offer valuable insights into patients’ daily activities, vital signs, and sleep patterns, several limitations of such data must be acknowledged. Wearable digital devices, while improving in precision, often have inconsistencies in data capture due to various factors such as device placement, user adherence, battery life, and other technical issues [12]. For example, step counts can vary significantly based on the make, model, and type of device (eg, smartphone vs Bluetooth digital pedometer vs smartwatch), and heart rate measurements can be affected by movement or improper device placement [13,14]. Heterogeneity, variability, and data quality are also known challenges with sleep data [15].

Another limitation of PGHD relates to its completeness and representativeness. The data that are in the PGHD database, for example, depend on what are available through an API from third-party digital health companies. Metadata that come from Apple Health may differ from what are available through Garmin or Fitbit. Additionally, not all individuals consistently use their devices and, in some cases, users may make unintentional entry errors (in cases of manual, self-entered PGHD) or intentionally alter data reporting. These behaviors can lead to gaps in data that can affect the continuity and reliability of health monitoring.

It can also be difficult to determine if a daily summary measurement (eg, daily step count) represents a full or partial day of use. Additionally, individuals who use mobile health apps and digital devices are likely not representative of broader patient populations, as users of mobile apps and digital devices tend to be younger, more health-conscious, and of higher socioeconomic status, or in other cases, have a higher burden of disease and health care needs. These trends can limit the generalizability of research findings generated from PGHD [16]. Finally, a key distinction between PGHD from wearables and devices and traditional health care data collected in clinical settings is that PGHD are tied to the device generating the data. As a result, there is some inherent uncertainty regarding the identity of the individual operating the device transmitting PGHD to the VHA.

Given these limitations, appropriately representing and analyzing PGHD are critical for health systems looking to integrate such data for clinical, research, and evaluation use. The lack of certain metadata from third-party health apps limits the ability to discern between older and newer makes and models of common wearables and other devices. Prior studies on older devices have raised concern about the performance in populations with chronic conditions and mobility issues [17]. As such, individuals and providers could become concerned (or reassured) about a patient’s status based on unreliable data from an unreliable device. In future iterations of the database, it is possible that more metadata from the third-party health aggregators (eg, make and model) will be available; however, availability will be dependent on what is available through the APIs, and may differ from one device or app to another.

These limitations necessitate careful consideration when interpreting PGHD and underscore the importance of complementing it with more traditional data obtained during clinical encounters. As a tool for monitoring health and wellness, consistent collection of PGHD can help monitor important trends over time and can help minimize the risk of any one individual data point being inaccurate.

## Potential Clinical Uses of the PGHD Database

**Figure 3.**
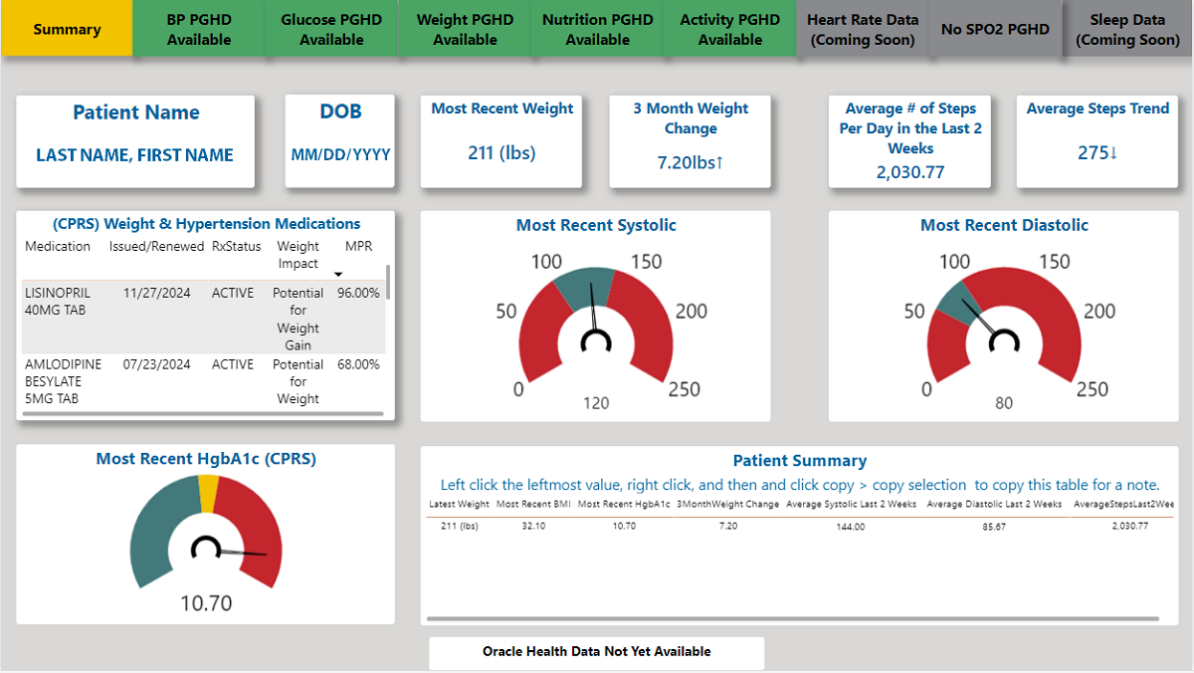
The Veterans Health Administration–Patient Generated Vitals and Health Data Dashboard—summary tab. BP: blood pressure; CPRS: computerized patient record system; DOB: date of birth; HgbA1c: hemoglobin A_1c_; MPR: medication possession ratio; PGHD: patient-generated health data.

The PGHD database establishes the infrastructure necessary to develop tools that support clinical use of PGHD. Currently, patients can view their own PGHD through the Share My Health Data mobile app’s daily dashboards and the VHA clinicians can view their patients’ PGHD through Power BI (Microsoft) clinical dashboards accessed through the VHA’s electronic health record and a provider-facing web application called Virtual Care Manager. These provider dashboards do not require additional logins but do require knowledge of where they are located within the electronic health record. These dashboards display PGHD alongside data from the patient’s medical record to facilitate the identification of trends and opportunities for clinical intervention. The Patient Generated Vitals and Health Data Dashboard contains tabs for different types of PGHD, such as blood pressure, blood glucose, weight, diet or nutrition, activity, and pulse oximetry data (Figures3  4). The Diabetes Dashboard combines PGHD (blood glucose, blood pressure, weight, nutrition, activity, and blood oxygen levels) with data collected during in-person visits, laboratory results, and active medication information.

**Figure 4.**
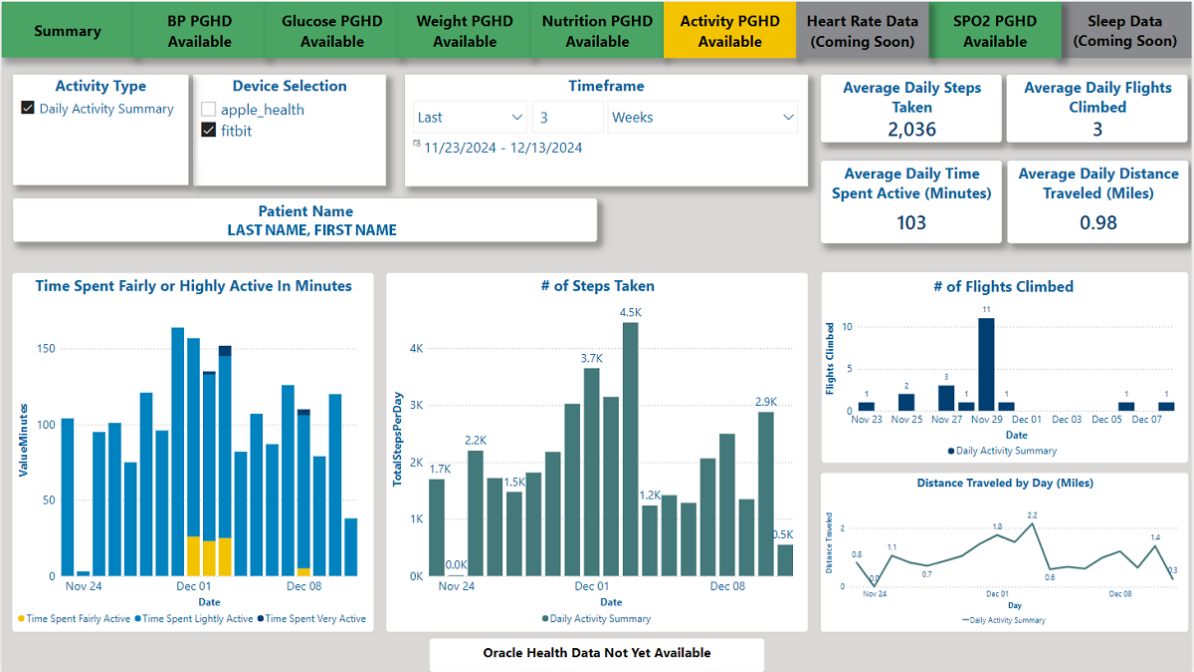
The Veterans Health Administration–Patient Generated Vitals and Health Data Dashboard—activity tab. BP: blood pressure; PGHD: patient-generated health data.

The Office of Connected Care is currently conducting pilot tests to assess the usability of PGHD in clinical care and the PGHD clinical dashboards. The pilot tests are designed to identify the level of effort required by veterans to sync and share their medical or activity devices, and to assess how clinical teams are incorporating PGHD into their clinical practice and workflows. Ongoing evaluations of the pilot tests will identify barriers and potential solutions to the integration of PGHD into clinical practice. Although the VHA has not yet committed to a set of metrics to define “success” for the expansion of PGHD, there is commitment to studying the lessons learned from the ongoing pilot studies to inform targets for growth.

## Potential Research and Evaluation Uses for the PGHD Database

The VHA has identified five priority areas for virtual care research, two of which directly relate to the VHA’s PGHD initiatives: (1) identifying implementation strategies to increase patient and clinician adoption of effective virtual care technologies, and (2) examining how best to use PGHD in combination with electronic health record data to generate clinically valuable alerts and predictions for providers [18]. The development of the Share My Health Data app and accompanying clinician dashboards will facilitate research and evaluation initiatives aligned with both of these priorities.

Identifying implementation strategies to increase patient and clinician adoption of effective virtual care technologies marks the beginning of an extensive research and evaluation effort to understand the value of PGHD. Specific areas of interest include the role of PGHD in facilitating patient engagement in treatment or self-management, shared decision-making, and population health management. The VHA’s PGHD-related research agenda also focuses on research to inform improvements to PGHD implementation and to evaluate the safety, effectiveness, and health equity of PGHD solutions [1]. Examples include studies that engage with clinicians to understand which types of PGHD should be prioritized for clinical use, the evaluation of practice protocols for PGHD, barriers and solutions to the interoperability of PGHD with the electronic health record, solutions to address disparities in access to PGHD, the impact of PGHD on patient outcomes and effectiveness of care, and the most effective ways to integrate PGHD into clinical workflows and decision-making processes.

The PGHD database can also be leveraged as part of research and evaluation initiatives designed to measure the impact of broader health care interventions. Doing so is critical to examining how best to use PGHD in combination with electronic health record data to generate clinically valuable alerts and predictions. For example, rather than relying on data captured in the electronic health record collected during clinical encounters, the Share My Health Data app can be used to gather real-world data in between clinical encounters to assess the effectiveness of interventions being tested such as continuous glucose monitoring data, digital home blood pressure cuff readings, sleep quality, or daily step counts from wearables.

The aforementioned research and evaluation priorities represent cutting-edge work in the field of health services research and technology-assisted care. Thus, the advent of the PGHD database represents an avenue to disrupt the field of health care delivery such that clinicians are able to adopt a more personalized and data-informed approach to treatment.

## Discussion

Both within and outside of the VHA, health care systems have been increasingly interested in exploring the potential value of PGHD. As personal digital devices that can seamlessly capture these data have become commonplace, the next era of health care will involve capturing and harnessing these rich data elements in patients’ day-to-day lives outside of the clinician’s office to facilitate delivering care that is informed by each patient’s unique digital phenotype. The advent of the Share My Health Data app and accompanying clinician dashboards represent an important step the VHA is taking to be on the cutting edge of this transformation. The extensive collection and thoughtful application of PGHD not only support the individual health journeys of veterans but can also contribute broadly to the efficiency and effectiveness of health care and service delivery.

The next step of the PGHD initiative at the VHA is focused on research and evaluation to identify how best to integrate PGHD into workflows and find where it provides the most value. The VHA is particularly interested in identifying high-priority clinical use cases for PGHD and understanding implementation strategies to encourage patient and provider adoption. On the provider end, evaluations of attitudes toward PGHD and barriers to integrating PGHD-driven insights into care are needed. On the patient end, work is needed to encourage engagement and better understand expectations around the use of PGHD. Veterans can currently access the PGHD that they share through the Share My Health Data app; plans exist for the development of rules and alerts, and an online platform where veterans can more fully interact with their data. Further, although the Share My Health Data app alerts users that their data may not be regularly monitored by their care teams, veterans may still expect their providers to review data prior to and during clinical appointments or to notify them of any concerns in between appointments. How to effectively communicate to veterans how PGHD will be used by their care teams is necessary to ensure that it aligns with veteran expectations and preferences.

As the PGHD infrastructure at the VHA evolves, developing and integrating clinical alerts and predictions that make care more proactive and individualized for patients is crucial. Certainly, in the digital phenotyping space, we have seen that important clinical occurrences such as mood episodes can be predicted with this type of PGHD [19,20], but we have yet to see the broad implementation of alerts or models of care that leverage these types of data in routine practice.

The future of health care at the VHA, underpinned by the expansive use of PGHD, looks toward not only achieving the broad goals of enhancing stakeholder experiences, improving population health and health equity, and reducing costs but also setting a benchmark for other US health care systems. The VHA is committed to the broad dissemination of research and operational findings related to the implementation of PGHD across its clinics and facilities nationwide. As the VHA pursues the next step of rollout for the Share My Health Data app, the emphasis will be on establishing clear ethical standards for using this data, identifying and addressing challenges presented by the data itself, and streamlining clinical workflows for implementation success. Already, numerous lessons have been learned that have yielded valuable insights. As the VHA’s PGHD infrastructure evolves, the VHA is committed to sharing these lessons broadly, along with accompanying recommendations. The hope is that these lessons will be beneficial for other health care systems and their own efforts to better integrate PGHD into health care delivery.

## Abbreviations

Annie: Annie for Veterans
API: application programming interface
FHIR: Fast Healthcare Interoperability Resources
JSON: JavaScript Object Notation
PGHD: patient-generated health data
VHA: Veterans Health Administration
